# Analyzing Mosquito (Diptera: Culicidae) Diversity in Pakistan by DNA Barcoding

**DOI:** 10.1371/journal.pone.0097268

**Published:** 2014-05-14

**Authors:** Muhammad Ashfaq, Paul D. N. Hebert, Jawwad H. Mirza, Arif M. Khan, Yusuf Zafar, M. Sajjad Mirza

**Affiliations:** 1 Biodiversity Institute of Ontario, University of Guelph, Guelph, ON, Canada; 2 National Institute for Biotechnology and Genetic Engineering, Jhang Road, Faisalabad, Pakistan; 3 Pakistan Atomic Energy Commission, Islamabad, Pakistan; Rosalind Franklin University, United States of America

## Abstract

**Background:**

Although they are important disease vectors mosquito biodiversity in Pakistan is poorly known. Recent epidemics of dengue fever have revealed the need for more detailed understanding of the diversity and distributions of mosquito species in this region. DNA barcoding improves the accuracy of mosquito inventories because morphological differences between many species are subtle, leading to misidentifications.

**Methodology/Principal Findings:**

Sequence variation in the barcode region of the mitochondrial COI gene was used to identify mosquito species, reveal genetic diversity, and map the distribution of the dengue-vector species in Pakistan. Analysis of 1684 mosquitoes from 491 sites in Punjab and Khyber Pakhtunkhwa during 2010–2013 revealed 32 species with the assemblage dominated by *Culex quinquefasciatus* (61% of the collection). The genus *Aedes* (*Stegomyia*) comprised 15% of the specimens, and was represented by six taxa with the two dengue vector species, *Ae. albopictus* and *Ae. aegypti*, dominant and broadly distributed. *Anopheles* made up another 6% of the catch with *An. subpictus* dominating. Barcode sequence divergence in conspecific specimens ranged from 0–2.4%, while congeneric species showed from 2.3–17.8% divergence. A global haplotype analysis of disease-vectors showed the presence of multiple haplotypes, although a single haplotype of each dengue-vector species was dominant in most countries. Geographic distribution of *Ae. aegypti* and *Ae. albopictus* showed the later species was dominant and found in both rural and urban environments.

**Conclusions:**

As the first DNA-based analysis of mosquitoes in Pakistan, this study has begun the construction of a barcode reference library for the mosquitoes of this region. Levels of genetic diversity varied among species. Because of its capacity to differentiate species, even those with subtle morphological differences, DNA barcoding aids accurate tracking of vector populations.

## Introduction

Mosquitoes are important vectors of animal diseases [1]. Although Pakistan is one of the hotspots for mosquito-vectored diseases [2], [3], mosquito biodiversity in the country is under-explored [4]. However, the recent outbreaks of dengue in Pakistan [5] have generated interest in mosquito distributions in this region [6]–[8]. Among the 3500 mosquito species recorded worldwide (www.mosquito-taxonomic-inventory.info) 104 have been documented from Pakistan and Bangladesh [4], but their morphological identification remains difficult.

Correct vector identification is very important to design strategies for managing vector-borne diseases [9]. Because detailed taxonomic studies have focused on mosquitoes that are vectors of human disease [10], other species have received little attention [11], [12]. Moreover, many closely related species of mosquitoes with differing ecological and host preferences are nearly indistinguishable morphologically [13]. These factors mean that the identification of mosquitoes to a species or sometimes even a genus is often difficult [14]–[16]. As a consequence, DNA-based approaches to mosquito identification [17]–[19], genetic diversity [20], [21], and molecular phylogeny [22], [23] have gained increasing adoption. Although use of nuclear genes is not uncommon [24]–[27], mitochondrial genes have gained primary adoption for analyzing genetic diversity in mosquitoes [28], [29]. DNA barcoding [30] has already seen frequent use for mosquitoes in varied contexts [31]–[37]. As a result, the overall DNA barcode library now includes records for 894 mosquito species among the 320K animal species which have been analyzed (www.boldsystems.org).

Prior studies have monitored mosquito populations using both morphological [38] and molecular approaches [39]. For example, Reisen et al. (1982) [40] used morphological identifications to assess the diversity of mosquitoes in Pakistan, especially those important in the transmission of viral diseases. Mousson et al. (2005) [41] subsequently used mitochondrial DNA variation to study the phylogeography and relationships of *Aedes aegypti* and *Aedes albopictus*, while Chen et al. (2002) [25] used 28S rDNA and COII to examine the distribution and vector status of *Anopheles minimus* in southern China. Correlation between vector genotypes and their capacity to transmit disease pathogens [42] has triggered interest in the genetic diversities of vector species [43]. In a study of three vector species (*Ae. aegypti*, *Ae. albopictus*, *Aedes vittatus*) in India, Angel & Joshi (2008) [44] found that infectivity of dengue virus varied both among species and regionally for a particular species. Studies by Pollitt et al. (2013) [45] and Anderson et al. (2004) [46], correlated mosquito strains/genotypes with their competence to transmit malaria parasites and La Crosse virus.

Studies on species composition and density of local mosquito populations have helped to develop better management strategies for mosquito-borne diseases [47],[48]. Such studies can establish a baseline of mosquito-borne virus activity allowing monitoring of change over time [49]. *Ae. aegypti* and *Ae. albopictus* have differing ecological preferences with the former species most prevalent in urbanized areas, while the latter is often common in rural settings [50], [51]. Although native to Southeast Asia, *Ae. albopictus* has extended its range [52] provoking concerns among disease management strategists [53]. The 2011 dengue epidemic in Pakistan showed considerable regional variation in severity, raising questions about the possible role of shifting distributions of its vector species as the cause.

Since the species checklist by Khan (1971) [4] which included mosquitoes from Pakistan and Bangladesh, only sporadic reports with limited scope have been completed [7], [40], [54]–[56]. Further, information on genetic diversity of regional mosquitoes usable for species assignments or to establish connections between local and the global mosquito fauna is either not available or is insufficient [8]. Although both *Ae. aegypti* and *Ae. albopictus* occur in Pakistan [4], there is little information on their relative abundance or distribution [57]. The current study employs DNA barcoding to identify, and analyze genetic diversity in mosquito species making it possible to map the distributions of dengue-vectors in the dengue-affected areas of Pakistan. The study also develops haplotype networks for the important disease vectors at a global scale connecting them in different regions.

## Materials and Methods

### Ethics statement

No specific permissions were required for this study. Mosquitoes from private residences were collected only after the consent of the respective owners. The study did not involve endangered or protected species.

### Collection of mosquitoes

**Figure 1:**
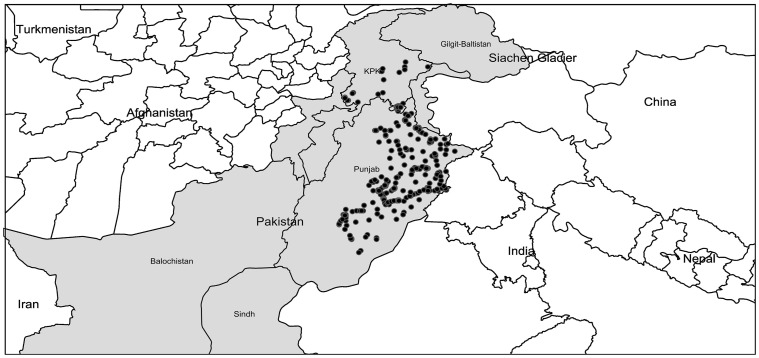
Map of collection localities (solid black dots) for mosquitoes in the dengue-affected areas of Punjab and the adjoining Khyber Pakhtunkhwa province.

Mosquitoes were collected at 450 urban and rural sites within Punjab, and from 41 locations in Khyber Pakhtunkhwa (KPK) during 2010–2013. These sites ranged in altitude from 92 m - 1004 m in Punjab and from 297 m–2376 m in KPK. Sampling sites included private residences, construction sites, junkyards, water catchments, marshes, ponds, and forests. Adults were collected with nets, aspirators, and light-traps coupled with a CO_2_ source, while larvae were sampled with pipettes and sieves. GPS coordinates were recorded (Table S1), and collection sites were mapped (Fig. 1) using (http://www.simplemappr.net). A total of 1942 specimens including 190 larvae were randomly chosen for DNA barcode analysis. Each mosquito was assigned a specimen number and photographed, and adults were identified using standard taxonomic keys [4], [40], [55], [58]. Species names follow those employed by the on-line resource (www.mosquito-taxonomic-inventory.info). Specimen data along with the collection information are accessible in the dataset DS-MAMOS (Barcoding Mosquitoes of Pakistan) on BOLD (www.boldsystems.org), the Barcode of Life Data System [59].

### DNA isolation, PCR amplification and sequencing

A single leg was removed from each adult specimen and transferred to a well pre-loaded with 30µl of 95% ethanol in a 96-well microplate, while larvae were processed using the protocol in Porco et al. (2010) [60]. DNA extraction, PCR amplification, and sequencing were performed at the Canadian Centre for DNA Barcoding (CCDB) following standard protocols [61]. Amplification of the COI-5′ barcode region was performed with primer pair C_LepFo1F (a cocktail of LepF1+LCO1490)/C_LepFo1R (a cocktail of LepR1+HCO2198) (http://www.dnabarcoding.ca/CCDB_DOCS/CCDB_PrimerSets.pdf) using the following PCR conditions: 94°C (1 min); 5 cycles of 94°C (30 s), 45°C (40 s), 72°C (1 min); 35 cycles of 94°C (30 s), 51°C (40 s), 72°C (1 min); and final extension of 72°C (10 min). PCRs were carried out in 12.5 µL reactions with a standard reaction cocktail and 2 µL of DNA template. PCR products were visualized on a 2% agarose E-gel 96 system (Invitrogen Inc.) and successful amplicons were bidirectionally sequenced using BigDye Terminator Cycle Sequencing Kit (v3.1) on an ABI 3730XL DNA Analyzer. The forward and the reverse sequences were assembled and aligned using CodonCode Aligner (CodonCode Corporation, USA). Sequences were subsequently translated in MEGA V5 [62] to verify that they were free of stop codons and gaps and uploaded onto BOLD. All sequences with their GenBank accession numbers (KF406349 to KF407931) are accessible in the dataset DS-MAMOS (Process IDs: MADIP255-10 to MADIP380-10; MADIP458-11 to MADIP473-11; MAMOS001-12 to MAMOS1425-12; MAMOS1521-13 to MAMOS2019-13).

### Data analysis

#### Species discrimination using DNA barcodes

The sequence from each specimen was compared to barcode sequences on GenBank using "Blast", and to sequences for 894 mosquito species on BOLD using the "Identification Request" function. Prior studies have revealed that most different species of Diptera show >2% sequence divergence at COI [63], and researchers have used a 2% distance threshold for species delimitation [64]. Based on reference barcode data, Ratnasingham & Hebert (2013) [65] have established the Barcode Index Number (BIN) system which assigns a unique global identifier to each sequence cluster. In most cases, specimens assigned to different BINs belong to different species, but the BIN system aids the organization of data for records lacking a formal taxonomic assignment. All mosquito sequences obtained in this study were assigned to a BIN.

#### Genetic diversity and phylogenetic analysis

Nucleotide sequences were aligned using ClustalW [66] in MEGA V5. The FASTA file was submitted to the online version of Automatic Barcode Gap Discovery (ABGD) [67] to generate distance histograms and distance ranks. The presence or absence of a “barcode gap” [68] was also determined for each species as a test of the reliability of its discrimination. Using the barcode gap criterion, a species is distinct from its nearest neighbor (NN) if its maximum intraspecific distance is less than the distance to its NN sequence. The "Barcode Gap Analysis" (BGA) was performed using BOLD. Genetic diversity indices and neutrality tests (Fu's *Fs* [69] and Tajima's *D* [70]) were performed in DnaSP v5.10.01 [71]. Calculations of Kimura 2-parameter (K2P) [72] genetic distances and NJ analysis were carried out using MEGA V5. Because most mosquito species were represented by multiple sequences, TAXONDNA was used to generate a consensus barcode for each species, enabling the generation of a compact tree [73]. A tree of all sequences is provided as (Fig. S1). The K2P distance model was used, along with pairwise deletion of missing sites to generate NJ trees, while support for tree nodes was estimated using 500 bootstrap replicates. Consensus sequences were used in Bayesian inference (BI) analysis and BI trees were obtained using MrBayes v3.2.0 [74] and the Markov Chain Monte Carlo (MCMC) technique. The data was partitioned in two ways: a single partition with parameters estimated across all codon positions, and a codon-partition in which each codon position was allowed different parameter estimates. All partitions were allowed a GTR + gamma model and analyses were run for 10 million generations by using four chains with sampling every 1,000 generations. Bayesian posterior probabilities were calculated from the sample points once the MCMC algorithm began to converge. The trees generated through this process were visualized using FigTree v1.4.0.

#### Haplotype and distribution analysis

Barcode sequences for important disease vectors (Ae. aegypti, Ae. albopictus, Anopheles subpictus, Anopheles stephensi, Anopheles peditaeniatus, Culex tritaeniorhynchus, Cx. quinquefasciatus) from Pakistan, were combined with published records from other countries and aligned in MEGA5 before being exported as MEGA files. Barcode haplotypes were determined using Arlequin v.3.5 [75]. For each species, a minimum spanning tree (MST) based on the number of nucleotide differences between haplotypes was constructed using a distance matrix from Arlequin in Hapstar v. 0.6 [76] to visualize the network of interrelationships between the haplotypes. The distributions of Ae. aegypti and Ae. albopictus were mapped using an online tool (www.simplemappr.net).

## Results

### Identification of mosquito species and DNA barcode analysis

**Figure 2:**
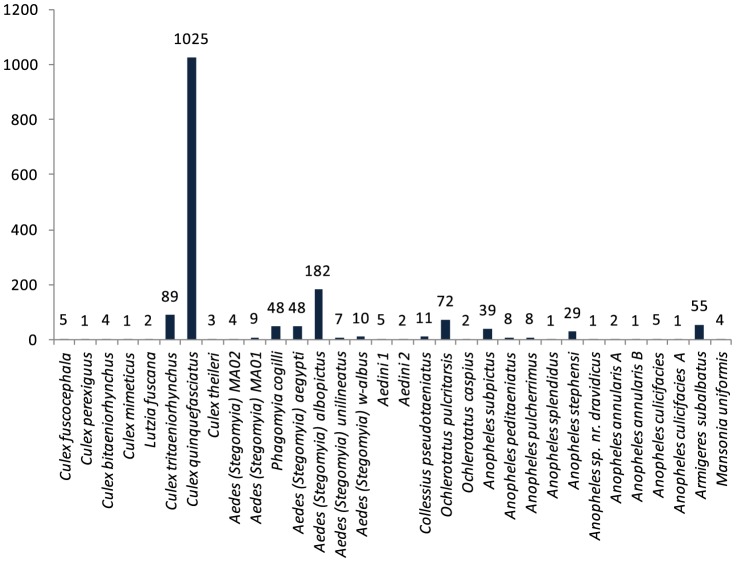
Mosquito species identified from the dengue-affected areas of Pakistan. The number of specimens of each species in the collection are indicated on the bars.

Morphological study indicated the presence of 21 mosquito species including four species of *Aedes* (*Stegomyia*) (*Ae. aegypti*, *Ae. albopictus*, *Ae. w-albus*, *Ae. unilineatus*), six species of Culex (*Cx. quinquefasciatus, Cx. theileri, Cx. tritaeniorhynchus, Cx. bitaeniorhynchus, Cx. mimeticus, Cx. fuscocephala*) and seven species of Anopheles (*An. subpictus, An. peditaeniatus, An. stephensi, An. splendidus, An. pulcherrimus, An. annularis, An. culicifacies*). Barcode sequences were recovered from 1684 of the 1942 specimens (87%). Fig. 2 shows the number of specimens of each species with a barcode sequence. Comparison with records in BOLD and GenBank revealed close sequence matches (<2% divergence) to 11 species which were not recognized morphologically. *Anopheles annularis* was partitioned into two taxa, *An. annularis A* and *An. annularis B*, while *An. culicifacies* was also found to include two sibling species, *An. culicifacies A* and a second unidentified taxon, raising the total to 23. Five more species (*Culex perexiguus*, *Phagomyia cogilli*, *Anopheles sp. nr. dravidicus, Ochlerotatus pulcritarsis, Ochlerotatus caspius*) were initially overlooked, but were identified through barcodes, raising the count to 28. Finally, two more species were only identifiable to a generic level (*Aedes* MA01, *Aedes* MA02), and two more to a tribe (Aedini 1, Aedini 2). The BIN system assigned these 32 taxa to 31 BINs; the sole case of a shared BIN involved *An. annularis A* and *An. annularis B.*

**Figure 3:**
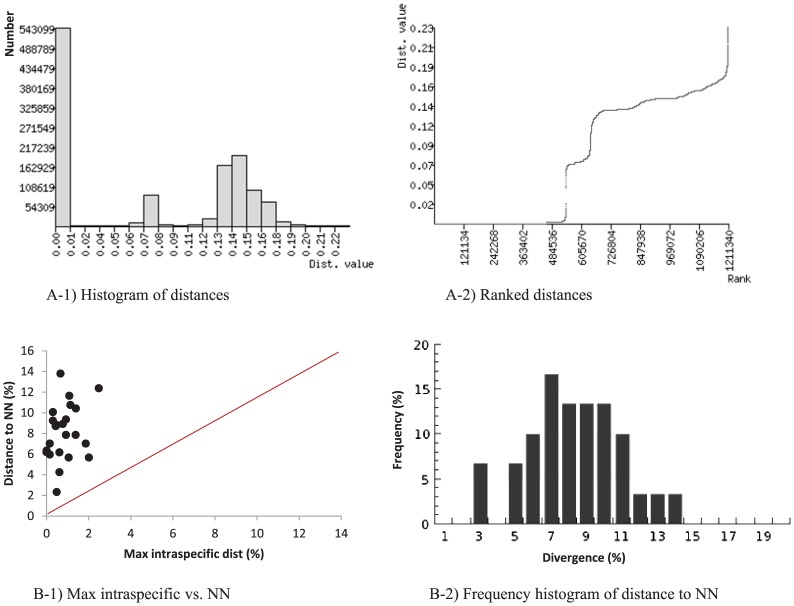
Pairwise distance divergence (%) (A) and barcode gap analysis (B) for mosquitoes from Punjab and Khyber Pakhtunkhwa as generated by ABGD [67] and by BOLD [59], respectively. NN  =  nearest neighbor.

The results of ABGD and BGA analyses revealed a clear gap between intraspecific and interspecific distances (Fig. 3). As well, the minimum distance to the nearest-neighbor (NN) was higher than the maximum intraspecific distance for every species (Fig. 3). In fact, NN distances exceeded 2.3% for all species (Fig. 3 B-1), and most ranged from 4.3–11% (Fig. 3 B-2). *An. culicifacies* was initially identified as a single species, but the barcode data revealed 4.3% divergence between two taxa; one was *An. culicifacies A*, but the other could not be identified because of the lack of reference barcode sequences for the other four known taxa in this complex. The barcode data also revealed that *An. annularis* included representatives of both *An. annularis A* and *An. annularis B*, species that were first recognized through polytene chromosome analysis [77]. Intraspecific distances could not be determined for the six species with just a single representative, but all of their NN distances were greater than 2.3%. Sequence divergence increased with taxonomic rank (Table 1) with little overlap between conspecific and congeneric distances. Intraspecific divergences ranged from 0.0–2.4% with a mean of 0.04%, while divergences for the species in a genus ranged from 2.3–17.8% with a mean of 8.2% (Table 1). Genetic diversity indices and results of neutrality tests for the barcodes are shown in Table 2. Average number of pairwise nucleotide differences (*k*), nucleotide diversity (π) and haplotype diversity (Hd) varied for the species. Fu's *Fs* and Tajima's *D* were significant for a majority of the species (Table 2).

**Table 1:** K2P sequence divergence at the COI barcode region among the mosquito species with >2 specimens, among the four genera with two or more species, and in the family Culicidae.

Distance class	*n*	Taxa	Comparisons	Min (%)	Mean (%)	Max (%)
Intraspecific	1638	24	543530	0	0.04	2.4
Congeners	1529	4	121341	2.3	8.1	17.8
Confamilial	1644	1	685675	6.4	14.5	22.5

**Table 2:** Genetic diversity indices and neutrality tests (Fu's *Fs* and Tajima's *D*) in the mtCOI-5′ (barcode) sequences of 21 mosquito species from Pakistan.

Species	*n*	S	k	π	h	Hd	Fu's *Fs*	Tajima's *D*
*Ae. aegypti*	48	5	0.27	0.0004	5	0.22	−3.95	−1.90
*Ae. albopictus*	182	6	0.43	0.0010	7	0.38	−3.88	−1.22
*Ae. unilineatus*	7	7	2.87	0.0055	5	0.86	−1.26	−1.03
*Ae. w-albus*	9	7	1.89	0.0031	5	0.80	−0.73	−1.00
*Aedes MA01*	9	13	4.83	0.0073	8	0.97	−2.82	0.05
*Aedes MA02*	4	6	3.00	0.0046	3	0.83	0.731	−0.81
Aedini 1	5	6	2.80	0.0043	4	0.90	−0.445	−0.19
*Ar. subalbatus*	54	3	0.27	0.0004	4	0.27	−2.17	−1.18
*An. culicifacies*	5	7	2.80	0.0042	5	1.00	−2.37	−1.16
*An. pulcherrimus*	8	2	0.50	0.0008	3	0.46	−0.99	−1.31
*An. peditaeniatus*	8	7	2.25	0.0034	6	0.93	−2.32	−0.79
*An. stephensi*	28	3	0.21	0.0005	4	0.21	−3.27	−1.73
*An. subpictus*	39	24	3.10	0.0052	17	0.87	−8.14	−1.84
*Co. pseudotaeniatus*	11	23	10.1	0.0154	6	0.89	1.59	0.72
*Cx. bitaeniorhynchus*	4	1	0.50	0.0008	2	0.50	0.17	−0.61
*Cx. fuscocephala*	5	4	2.00	0.0030	4	0.90	−1.01	0.27
*Cx. quinquefasciatus*	1018	23	0.09	0.0002	24	0.09	−64.4	−2.31
*Cx. tritaeniorhynchus*	89	33	2.78	0.0057	33	0.90	−27.08	−1.88
*Ma. uniformis*	4	4	2.33	0.0038	4	1.00	−1.62	0.65
*Oc. pulcritarsis*	72	32	4.74	0.0076	37	0.96	−25.58	−1.04
*Ph. cogilli*	48	19	3.16	0.0074	18	0.89	−7.36	−0.86

*n*: number of sequences; S: number of polymorphic sites; k: average number of pairwise nucleotide differences; π: nucleotide diversity; h; number of haplotypes; Hd: haplotype diversity.

Fu's *Fs:* A negative value of *F_S_* is evidence for an excess number of alleles, as would be expected from a recent population expansion or from genetic hitchhiking. Statistical significance: Not significant, P>0.02.

Tajima's *D*: A negative Tajima's *D* signifies an excess of low frequency polymorphisms relative to expectation. Statistical significance: Not significant, P>0.10.

Species represented by <3 specimens or barcodes with <500 bp were not included in the analyses.

**Figure 4:**
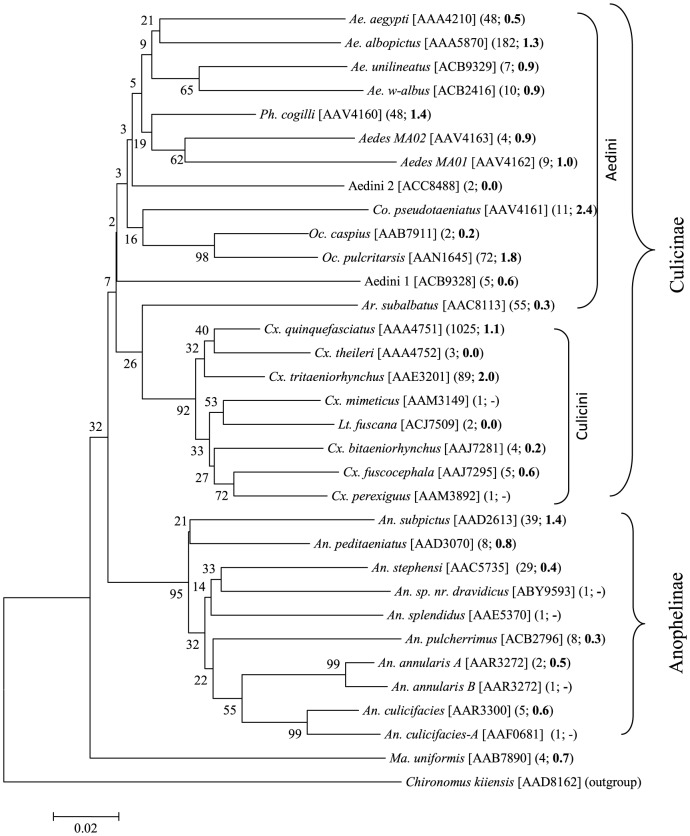
NJ analysis of mosquitoes collected from Punjab and Khyber Pakhtunkhwa. Bootstrap values (500 replicates) are shown above the branches. The scale bar shows K2P distances. Barcode Index Numbers (BINs) follow the species name in square brackets and the number of sequences analyzed and the intraspecific K2P distances (in bold) are included in parenthesis. Analyses were conducted in MEGA5.

**Figure 5:**
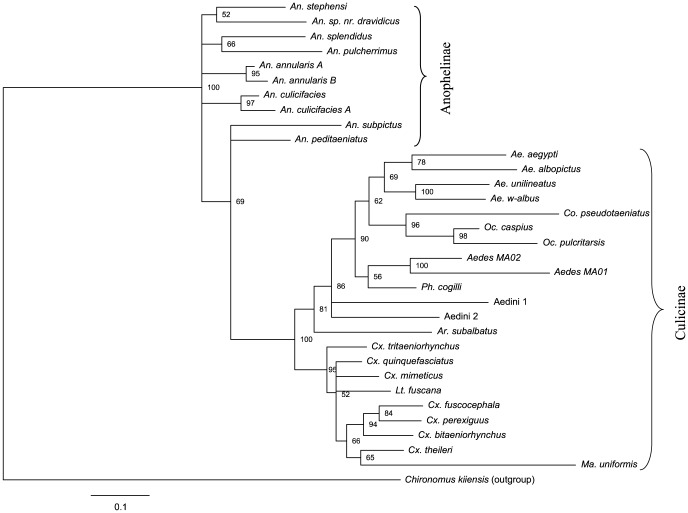
Phylogenetic tree of mosquito species estimated using Bayesian inference and the codon partitioned analysis. Posterior probability shown at nodes.

NJ analysis showed that the representatives of each species formed a monophyletic cluster (Fig. 4). The maximum intraspecific distance for *Cx. quinquefasciatus* (*n* = 1025) was 1.1%, while the distances for the two dengue vectors, *Ae. aegypti* (*n* = 48) and *Ae. albopictus* (*n* = 182) were 0.5% and 1.3%, respectively (Fig. 5). Among Anophelini, *An. subpictus* (*n* = 39) showed the highest intraspecific distance (1.4%). The only species with >2% intraspecific distance was *Collessius pseudotaeniatus* (2.4%). Species from the three mosquito tribes (Aedini, Anophelini, Culicini) mostly clustered with other members of their tribe. The cryptic species pair, *An. annularis A* and *An. annularis B* shared the same BIN, but their nodes were separated by a 99% bootstrap value in the NJ tree (Fig. 4). A phylogenetic tree of mosquito species estimated using Bayesian inference is presented in Fig. 5. The overall node pattern of the phylogenetic tree was similar to that of NJ tree, other than a close relationship between Aedini and Culicini was more evident and all the species branched with their respective subfamilies and tribes. The posterior probability values for all the nodes were greater than 50%.

### Global haplotype diversity and distributions of dengue vector species in Pakistan

**Figure 6:**
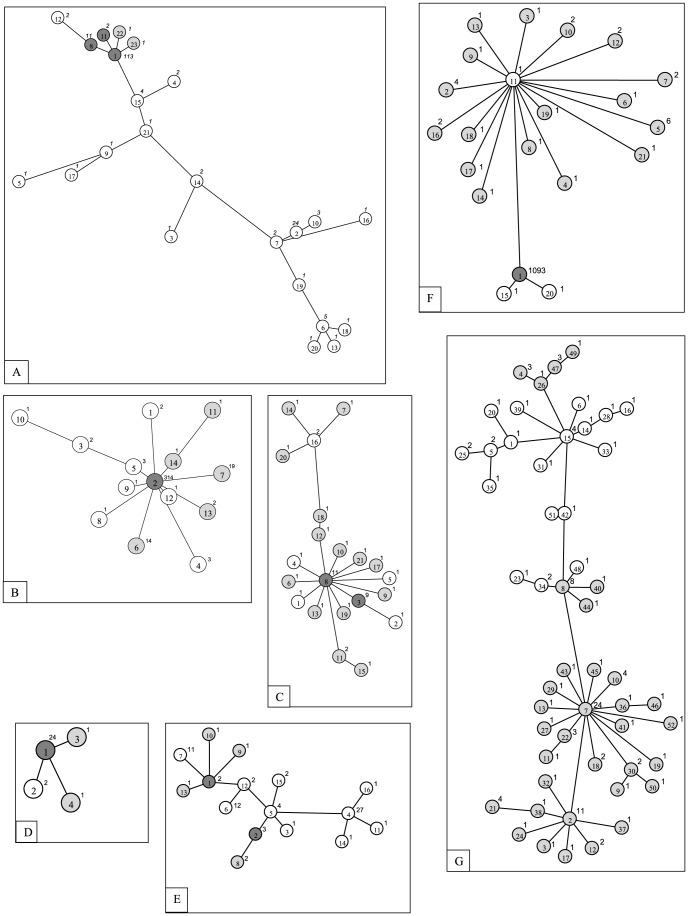
Barcode haplotype networks of vector mosquitoes from Pakistan. Haplotype number and frequency is indicated inside and besides the corresponding circle, respectively. Haplotypes shared between Pakistan and other countries, found solely in Pakistan, and not found in Pakistan are indicated by dark grey, light grey, and blank circles, respectively. Haplotypes (in brackets) and their origin countries follow the species below (except for haplotypes exclusively from Pakistan indicated in light grey). A) *Aedes aegypti:* (1) Argentina, Australia, Bolivia, Brazil, Cambodia, Canada, Chile, France, French Polynesia, Gabon, Guinea, India, Laos, Pakistan, Russia, Thailand, Uganda, USA, Venezuela, Vietnam; (2) Brazil, Cambodia, Canada, Laos, Martinique, Thailand, USA; (3) South Africa; (4) Canada, USA; (5) (21) Cote d'Ivoire; (6) Australia, Bolivia, Martinique; (7) Martinique, Mexico; (8) Australia, Cambodia, Pakistan, Thailand; (9) Mexico; (10) UK, USA; (11) Bolivia, Pakistan; (12) India, Vietnam; (13) (18) (20) Bolivia; (14) Vietnam; (15) Cameroon, Cote d'Ivoire, Guinea; (16) Tanzania; (17) Australia; (19) Europa Island. B) *Aedes albopictus:* (1) Thailand; (2) Brazil, France, Germany, Greece, Italy, Japan, Lebanon, Madagascar, Pakistan, Re Union: La possession, Russia, Thailand, Turkey, USA, Hawaii (USA), Vietnam; (3)Romania; (4) Australia, Taiwan; (5) Germany; (8) Cambodia; (9) Madagascar; (10) India; (12) Vietnam. C) *Anopheles subpictus:* (1) (2) (4) (5) (16) India; (3) (8) India, Pakistan. D) *Anopheles stephensi:* (1) Pakistan, South Africa; (2) Thailand. E) *Anopheles peditaeniatus:* (1) (2) India, Pakistan; (3) India; (4) (5) (6) (7) (11) (12) (14) (15) (16) Thailand. F) *Culex quinquefasciatus:* (1) Brazil, China, India, Iran, Japan, Malaysia, Mexico, Pakistan, Thailand, Uganda, USA; (11) (20) Brazil; (15) Mexico. G) *Culex tritaeniorhynchus:* (1) (14) (23) (34) (25) (28) (33) (35) (42) (48) (51) Japan; (6) (16) (31) (39) China; (5) (15) China, Japan; (20) Thailand.

The sequence data for seven disease vector mosquitoes from Pakistan were placed in a broader perspective by including barcode results from other regions. When data (*n* = 182) from 29 nations was considered for *Ae. aegypti*, maximum sequence divergence reached 2.2% (mean = 0.5%),while the maximum distance for *Ae. albopictus* (*n* = 365) from 21 countries was 1.9% (mean  =  0.3%). Twenty three haplotypes were detected in *Ae. aegypti* although one was dominant (62%), occuring in 20 countries including Pakistan (Fig. 6A). Eighteen of these 23 haplotypes were not detected in Pakistan, while two were only detected there (Fig. 6A). *Ae. albopictus* showed the presence of 14 haplotypes, one being in abundance (86%) and present in 16 countries (Fig. 6B). Eight of these 14 haplotypes were not detected in Pakistan, while five were only reported from there (Fig. 6B). The malaria vectors, *An. subpictus* (*n* = 41; max dist 2.2%) and *An. stephensi* (*n* = 28; max dist 0.4%) showed the presence of 21 and 4 haplotypes, respectively (Fig. 6C,D). The most common haplotype of *An. stephensi* was detected in Pakistan and South Africa (GU908046). There were 16 haplotypes for *An. peditaeniatus* (*n* = 72; max dist 1.8%); ten were detected exclusively in Thailand, four in Pakistan and the remaining two were shared between India and Pakistan (Fig. 6E). Barcodes of *Cx. quinquefasciatus* (*n* = 1125; max dist 1.2%) from 11 countries revealed 21 haplotypes, 17 of them were found solely in Pakistan, three were not detected in Pakistan, and one most frequent haplotype in Pakistan was also found in 10 other countries (Fig. 6F). *Cx. tritaeniorhynchus* from four countries (*n* = 113; max dist 2.3%) showed the presence of 52 barcode haplotypes and 34 were exclusively from Pakistan (Fig. 6G).

**Figure 7:**
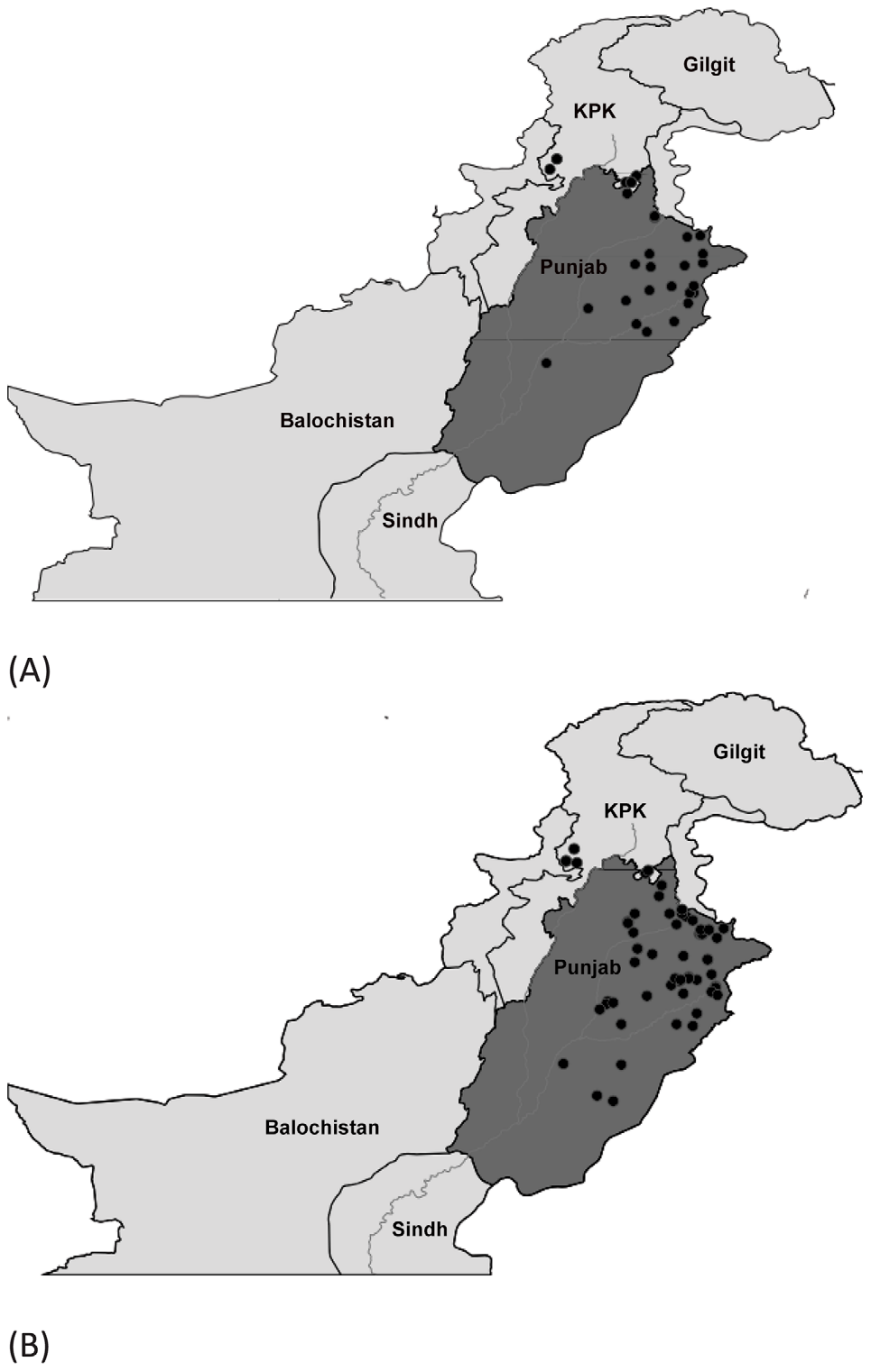
Map showing the distribution of *Aedes (Stegomyia) aegypti* (A) and *Aedes (Stegomyia) albopictus* (B) in the dengue-affected areas of Punjab, Pakistan.

Both *Ae. aegypti* and *Ae. albopictus* were present in almost all the dengue affected districts of Punjab (Fig. 7A,B). Although *Ae. albopictus* was detected from more locations, both species were detected in the urban areas of central Punjab most impacted by dengue. *Ae. aegypti* was collected at sites ranging in elevation from 112 m–1004 m, while *Ae. albopictus* had a slightly narrower elevational range (110 m−672 m). *Anopheles* and *Culex* species were present in all the areas included in the study with *Cx. quinquefasciatus* and *An. subpictus* the most frequent members of their respective genera (distributional data not shown).

## Discussion

The mosquito fauna of South Asia is known to be diverse; 104 species have been reported from Pakistan and Bangladesh [4]. Local surveys have reported fewer taxa, including 43 species from Punjab [54], 30 species from the Changa Manga National Forest in central Punjab [40], and 21 species from the Swat valley in an adjoining province [7]. *Cx. quinquefasciatus* was the most abundant species in our study, mirroring results from earlier work in Swat [7], but prior studies in Punjab indicated the dominance of *Cx. tritaeniorhynchus* [40], [54]. Although *Ae. albopictus* was the dominant species of *Aedes* in Punjab, it was not detected at higher elevation sites (1100 m) in Swat [7]. Interestingly, Reisen et al. (1982) [40] found that *Aedes lineatopennis* was the most abundant species of *Aedes*, suggesting a shift in species composition through time. Although *Ae. albopictus* occurred at sites as high as 1200 m on La Reunion [78], it was limited to sites with an elevation less than 700 m in Pakistan. However, other species, such as *Oc. pulcritarsis* showed a much broader elevational range (111 m−2376 m).

Because of the difficulty of morphology-based identifications in mosquitoes, DNA-based approaches have gained increasing usage. The analysis of sequence variation in the internal transcribed spacer 2 (ITS2) revealed sibling species in both the *Anopheles maculipennis* [24] and *Anopheles crucians* [79] complexes. Atrie et al. (1999) [77] detected two sibling species (A and B) in the *An. annularis* complex through cytogenetic analysis, and subsequent work revealed their discrimination by ribosomal DNA [26], results extended by our analyses which revealed their 2.3% divergence at COI. Kumar et al. (2007) [31] found that DNA barcodes reliably identified 62 of 63 mosquito species from India; *Ochlerotatus portonovoensis* and *Ochlerotatus wardi* were the only two species which could not be discriminated. However, Kumar et al. (2013) [35] also concluded that Indian populations of *Anopheles fluviatilis* previously assigned to a complex of three species (S, T, U) were actually conspecific. Although there are rare cases of failure, the overall success in discrimination of species underscores the importance of constructing a DNA barcode reference library for all Culicidae.

The presence of endemics in South Asia [80], [81] reinforces the importance of developing regional barcode libraries to reveal overlooked species. The mosquito species reported in two major studies from India [31] and China [34] show just 50% overlap with the species assemblage in this study, making it clear that many more species await analysis. The completion of this task is complicated by the difficulty in identifying some taxa, especially those in the tribe Aedini. Species of *Aedes* are a particular challenge [15], with recurrent taxonomic revisions indicating the ongoing controversy [14]–[16]. This uncertainty in the application of names provides an incentive to consider alternate approaches for species discrimination based on the recognition of DNA sequence clusters. The possible effectiveness of this approach is signaled by the fact that the species examined in this study showed average intraspecific distances ranging from 0−2.4%, while distances between congeneric species varied from 2.3−17.8%. Wang et al. (2012) [34] reported similar levels of conspecific (0−1.67%) and congeneric (2.3−21.8%) divergences in their study of 122 mosquito species in China. The recently established BIN system has created a permanent registry for DNA barcode clusters [65], and the present study provides an opportunity to test the correspondence between the clusters recognized by it and the mosquito species recognized in our study through morphological analysis and examination of the NJ and phylogenetic trees and NN distances. This check indicated near perfect congruence as the 32 species were assigned to 31 BINs; the sole merger involved the sibling species, *An. annularis A* and *An. annularis B*. Similarly, all 43 mosquito species analyzed by Kumar et al. (2007) [31] with adequate sequence data for a BIN assignment were placed in a unique BIN.

The importance of sequence analysis is clear in situations where one member of sibling species complex is a vector and the other is not. For example, *An. annularis A* transmits malaria in some parts of India, but *An. annularis B* does not [77]. Aside from enabling the discrimination of sibling taxa, DNA barcoding can reveal variation in single species which may have implications for disease transmission [82]. Genetic diversity indices showed a higher genetic divergence in some species and not in others. For example, *k* and π values were higher in *Cx. tritaeniorhynchus* and lower in *Cx. quinquefasciatus*. Intraspecific diversity also varied in the species of *Aedes* and *Anopheles*. A recent study on *Cx. quinquefasciatus* from Malaysia [20] has reported a low COI diversity for this species, and our results are in agreement with that study. But for the same species from India, Sharma et al. (2010) [83] have reported a high diversity in 16S rRNA. This incongruence may support the ongoing debate on mitochondrial and nuclear discordance in animals [84]. Prior work has revealed substantial genetic diversities in both *Ae. aegypti* and *Ae. albopictus*, providing important clues on population relationships and origins [85], [86] and insights into their role in the transmission of dengue [41]. Both species showed considerable barcode variation within Pakistan, but *Ae. albopictus* was less variable than *Ae. aegypti* when analysis considered the entire range of each species, a result congruent with Mousson et al. (2005) [43]. However, we did not find any evidence of genetic differentiation between specimens from rural and urban habitats or between larval and adult stages although the latter test was weak because larvae comprised just 7% of the specimens (122/1684). Our study revealed a single globally dominant COI haplotype in *Ae. aegypti,* while Moore et al. (2013) [36] found two major ND4 haplotypes in its African populations. Using exon primed intron crossing and microsatellite markers, Olanratmanee et al. (2013) [87] reported two genetic clusters in one region of Thailand and considered the implications of this diversity for a dengue suppression strategy. Mousson et al. (2005) [41] found little sequence variation in three mitochondrial genes for *Ae. albopictus* from 15 countries on five continents. Our analysis also revealed one major COI lineage in *Ae. albopictus*, widely distributed in 16 countries.

The results of our survey indicated that *Ae. albopictus* was more widely distributed and commoner than *Ae. aegypti* in Punjab. Akhtar et al. (2012) [57] found that larvae of *Ae. aegypti* predominated (65%) in collections from water-pots inside houses in Lahore during 2011. Although, the current dominance of *Ae. albopictus* in Pakistan supports a trend towards expansion of *Ae. albopictus* and a decline of *Ae. aegypti* in many areas of the world [88], [89], effect of sampling method on the variation in results cannot be ruled out.

In conclusion, this study has provided baseline data on composition and genetic diversity in the mosquito fauna of Pakistan, information that should be useful in tracking mosquito-borne diseases. The prevalence of *Ae. albopictus* in urban areas may suggest its importance in the spread of dengue. Because DNA barcoding can resolve cryptic mosquito species and identify their immature stages [90], it provides a valuable tool for large-scale vector identification and disease surveillance programs.

## Supporting Information

Figure S1 **NJ analysis of mosquitoes collected from dengue-affected areas of Punjab and adjoining Khyber Pakhtunkhwa.** Bootstrap values (500 replicates) are shown above the branches. Species names are preceded by the specimen Process IDs (Barcode of Life Data Systems). The scale bar shows K2P distances. *Chironomus kiiensis* (Diptera: Chironomidae) was included as an outgroup. Analyses were conducted in MEGA5.
(PS) Click here for additional data file.

Table S1 **GPS coordinates of mosquito collection sites in Pakistan.**
(XLS)
